# Intact gastro-intestinal tract removal from pig carcasses in a novel Meat Factory Cell approach

**DOI:** 10.1186/s13028-020-00546-y

**Published:** 2020-08-31

**Authors:** Ole Alvseike, Miguel Prieto, Per Håkon Bjørnstad, Alex Mason

**Affiliations:** 1 Animalia – Norwegian Meat and Poultry Research Center, P.O. Box 396 Økern, 0513 Oslo, Norway; 2 Department of Hygiene and Technology of Foods, Veterinary Faculty, University of León, 24071 León, Spain; 3 Institute of Food Science and Technology, University of León, 24071 León, Spain; 4 Faculty of Science and Technology, Norwegian University of Life Sciences, 1430 Ås, Norway

**Keywords:** Evisceration, Meat inspection, Pork, Slaughter hygiene

## Abstract

Conventional automated slaughter lines for pigs are organised as disassembly lines with many specialised machines. High costs and capacities make them relevant only for large scale meat production. The ambition with the novel Meat Factory Cell (MFC) concept is to provide the meat industry with a robust and flexible automation platform that is also relevant for smaller scale production. The MFC process deviates radically from conventional processing of pig carcasses after singeing. In MFC, the limbs are removed first. Then the dorsal muscles along the spinal axis from tail to head are removed with the column and rind in one meat cut, followed by removal of the viscera. Finally, the cut ribs and belly are removed. Such approaches to automation in pig abattoirs and cutting plants are highly needed in smaller scale production, and they should produce meat and offal as hygienically as conventional factories. This case study reports the evisceration of 37 pigs in 9 trials performed in 2019. Several approaches were tested with a prototype carcass holding unit. Evisceration could be undertaken without the need to cut through the gastrointestinal tract from tongue to rectum, reducing the probability of accidental faecal contamination of pork carcasses from the gut content. The Meat Factory Cell procedure is an advance towards automated evisceration of pig carcasses which is both simple and hygienic. The traditional separation of internal organs into a pluck set and a set of stomach and bowels was more prone to leakages.

## Findings

The Meat Factory Cell (MFC) approach for pig slaughter and primary cutting has recently been suggested and described [1, 2]. MFC applies three principal changes to conventional meat production and processing:

1. Work partly organised in cell stations instead of lines.
2. Combine and merge elements of slaughter and meat primal cutting.
3. “Disassemble” the carcass from outside-in, without removal of internal organs before removal of most primary cuts.

Alternative processes should at least be as hygienic as traditional slaughter and cutting. Improved hygiene is expected from the MFC concept as the meaty limbs, neck and loin are removed first. These primary cuts are not subject to faecal contamination from intestinal content. The MFC process results in seven cuts; Four limbs, the saddle including head and tail, the viscera including pluck, stomach and intestines, and a cut comprised of ribs and belly, i.e. the belly-cut. Also, from a meat inspection point of view, detection of lesions and abnormalities should at least be as sensitive as in traditional procedures [2].

Avoiding bacterial contamination of carcasses and meat is the most important hygienic challenge in meat industry. Evisceration carries a high probability of carcass contamination due to knife cuts and perforations resulting in leakage of the intestinal content. Good Hygiene Practise for evisceration includes ensuring that the probability of perforating the viscera, alimentary tract, uterus, urinary bladder, and gall bladder is minimised during separation cuts [3]. In addition, and regardless of accidental knife perforations, the two ends of the gastrointestinal tract are potential sources of carcass contamination [3]. In conventional slaughter lines, the pig carcasses are suspended by the hind legs. This allows use “bagging”, a technique in which a plastic bag is used to seal the rectum after circum-anal incision [4]. The sealed rectum is then pulled through the transected *Os pubis* and removed together with the intestines.

The oral cavity, with the tongue and palatine tonsils, is known to harbour a high bacterial load, including zoonotic bacteria [5]. It has been considered optimal to avoid cutting into tonsils while removal of the pluck, and “stunning, bleeding, skinning, evisceration and other dressing must be carried out without undue delay and in a manner that avoids contaminating the meat. In particular, the trachea and oesophagus must remain intact during bleeding” [6]. However, recently an alternative approach leaving the tongue with tonsils, pharynx, larynx and part of trachea and oesophagus intact on the head produced the lowest contamination levels on pig carcasses [7].

According to the Regulation EC No 853/2004, Annex III, Section I, Chapter IV, point 7 c, “measures must be taken to prevent the spillage of digestive tract content during and after evisceration” [6]. It was hypothesised that the MFC approach could possibly allow the alimentary tract to be removed intact [2]. If successful, this approach would significantly improve hygiene and reduce zoonotic risks associated with pork. We are not aware that removal of the entire gastrointestinal tract has been reported before and so, probably for the first time, fulfilling this intention of the EU legislation.

The aim of this study was to test in practice and describe evisceration of gastro-intestinal tract at slaughter of pigs in the MFC approach in a manner feasible for automation.

**Fig. 1:**
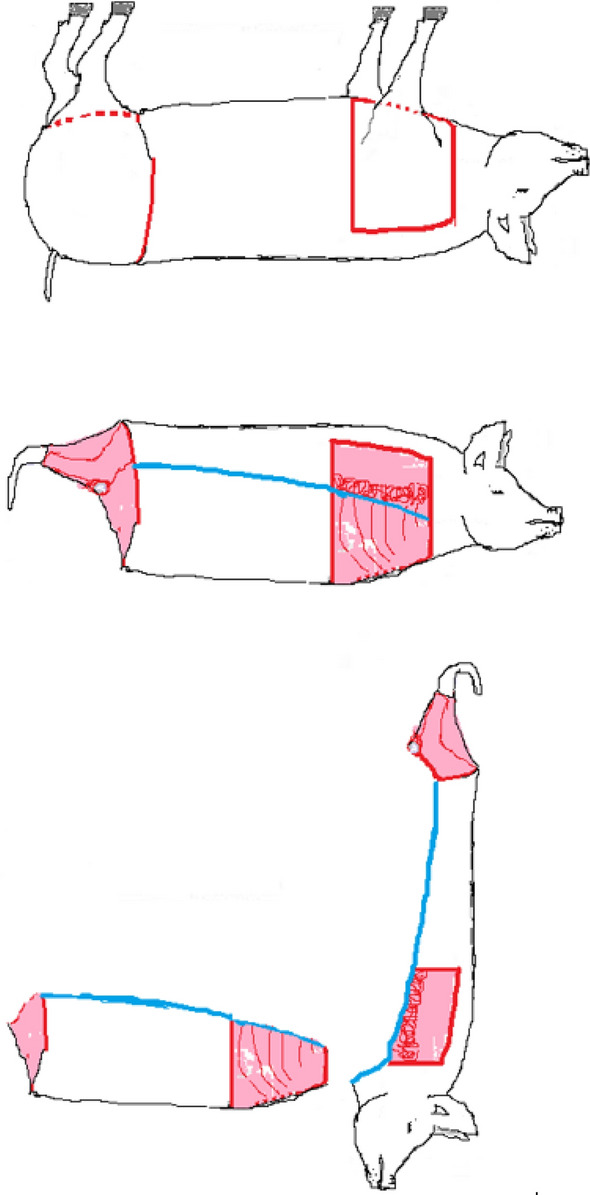
Meat Factory Cell schematic cutting pattern. The Meat Factory process deviates from conventional slaughter and cutting processes. After the pig carcass has been scalded and singed, the primary cutting is undertaken when the carcass is still warm. Then, the forelimbs and hindlimbs are removed and brought out of the cell to a rack. Then the body, (*truncus*), is turned 180° around its longitudinal axis. In front of the coxa wing, the soft belly wall is punctured and a cut moving cranially is made laterally to the loin (*M. longissimus dorsi*) (blue line). The saddle (hip bone, loins, neck and head) is then lifted up and the viscera loosened from mesentery ventral to the spine (*columna*)

Figure 1 shows the schematic cutting pattern applied in the trials. Five pigs where processed in three iterative trials conducted at a workshop on a farm in Norway from April to June 2019. After three trials (5 pigs), it was concluded how the carcass should be fixed, tools of choice, cutting trajectories and a more detailed procedure. Then, 32 crossbred white pigs, castrates and females, with approximately 110 kg live weight were slaughtered over a 6-week period in an abattoir, in total 37 pigs.

The first five pigs were stunned with a captive bolt pistol and bled indoors at the piggery, transported with tractor to the scalding bath, scalded in a hot water bath (approx. 65 °C), scraped manually and singed with a gas burner.

**Fig. 2:**
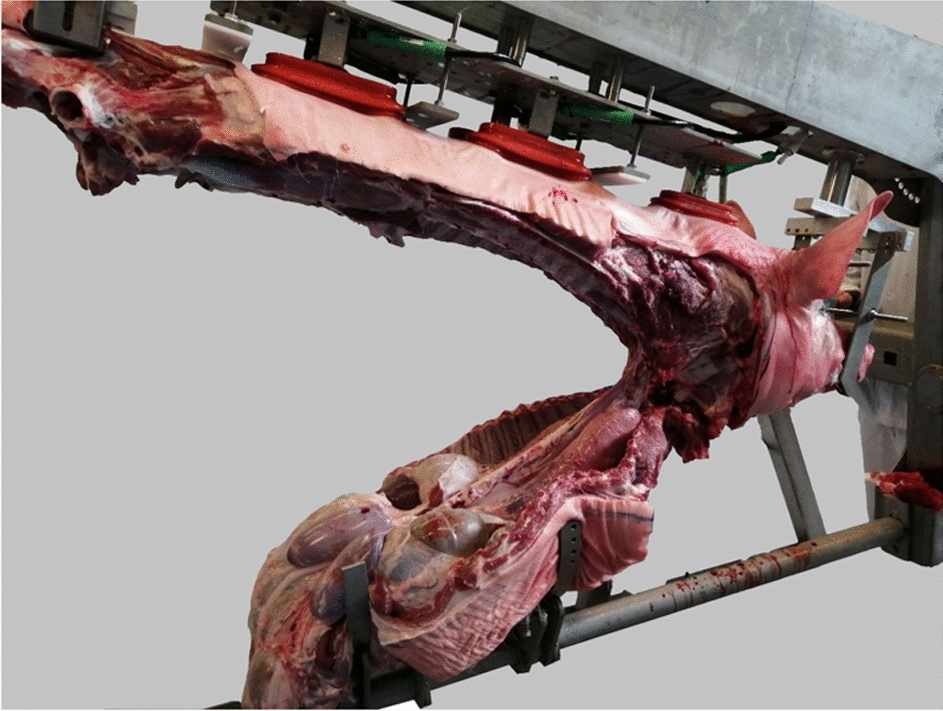
The Meat Factory Cell’s “Carcass Handling Unit”. The limbs have been removed and the belly and ribs sawed approximately 12–15 cm from the spine. Then the truncus has been lifted up. Trachea, oesophagus with some soft tissue is available for the butcher

The pigs were bagged and brought to the MFC after singeing and mounted to a prototype Carcass Holding Unit (CHU) (Tronrud Engineering AS, Hønefoss, Norway) designed for the MFC approach. The system was designed to accept a carcass from the hanging position (e.g. vertically from a rail) and present horizontally for cutting. The carcass could also be rotated 180 degrees—cutting began with the belly-cut facing upwards and proceeded to belly-cut facing downwards once all limbs had been removed. The carcass was fixed at the head (snout and eye sockets) as well as at the rear. A serrated clamp fixed *Os sacrum* after removal of the hind limbs. Vacuum suction cups (n = 5) held the back of the pig to enable initial fixation, stretching during rib sawing, and lifting and removal of the back prior to internal organ removal (Fig. 2).

The slaughter and cutting process was developed iteratively in collaboration with veterinarians, butchers and engineers. Limbs were removed with a knife, and the entire back from hip to neck lifted away as shown in Fig. 2. Then the thoracic and abdominal viscera were exposed, resting on the ribs and sides as shown in Fig. 2. The butcher freed the trachea and oesophagus towards the larynx. With two lateral cuts along the mandibles, avoiding the tonsils, and a transverse cut through the soft palate it was possible to remove the tongue as well as the pharynx with tonsils. While gripping firmly around trachea and oesophagus beneath larynx, the organs where pulled backwards.

Three alternative principal evisceration processes for the pluck were identified:

1. Removal of tongue, larynx, oesophagus, trachea, lungs and heart in one piece. This necessitates a cut through oesophagus close to the diaphragm.
2. Similar to 1, but without cutting the oesophagus. Instead, the diaphragm was loosened with a knife at the attachment to the thoracic and abdominal wall. The viscera were continuously pulled in the aboral direction, loosening mesenteries and normal adherences with a knife until they detached from the caudal end of the belly-cut. The easiest operation was to include the peritoneum and underlying fats (flare fat) in the removed viscera. As a result, the entire gastro-intestinal tract and other viscera (tongue, pharynx, tonsils, oesophagus, stomach, liver, gall bladder, mesenteries, flare fat, urinary bladder, genitalia and intestines from duodenum to anus) were removed in one piece. Heart, lungs and spleen followed the set, while the kidneys could either follow or be left on the back to ease meat inspection. The entire set of viscera is shown in Fig. 3.

**Fig. 3:**
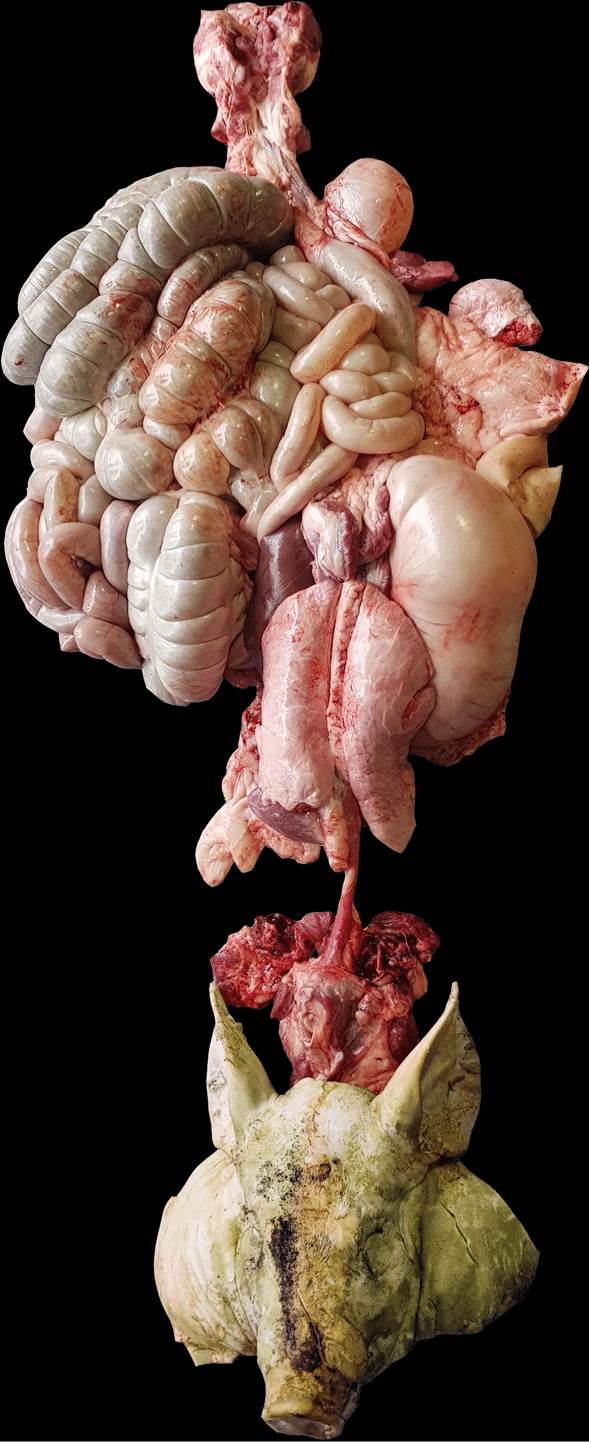
Evisceration in a Meat Factory Cell. The picture shows the gastrointestinal tract intact in one piece. Here, the kidneys were left on the back part. The organs were photographed lying on the floor
3. Similar to 2, but a conventional set of plucks was produced by transecting oesophagus at the entrance to the stomach. The resulting pluck consisted of tongue, trachea, lungs, heart, diaphragm and liver. The stomach and intestines then glided off or could be physically torn off including the flare fat.

Figure 2 shows that trachea and oesophagus are being openly exposed. The recently described alternative pluck removal [7] was not explored but would probably further simplify robotic operations within the cell.

Our study reports the methodology and experiences with alternative evisceration methods applied in MFC pilots. Most interestingly, the alternative procedure (2) allowed the butcher to remove the gastrointestinal tract intact in one piece. In fact, the presentation of the carcass made it a relatively easy technique for the butcher. The traditional separation of internal organs into a pluck set and a set of stomach and bowels was more cumbersome with higher probability of ruptures and leakages.

From a veterinary hygienic perspective, we have not so far identified disadvantages with the MFC approach. The biggest challenge is that MFC does not fit with the wording of regulatory paragraphs, e.g. that the viscera should be removed as soon as possible [6]. In MFC, evisceration is almost the last operation, but the whole process is undertaken within 8–12 min in the present demo set-up.

Coincidentally, two pigs presented pathological changes. One had an umbilical hernia filled with greyish debris and turbid fluid. The orifice to the abdominal cavity was practically closed by fat tissues. The flare fat was easily removed, and local condemnation of affected tissue was hygienically undertaken. The second presented chronic fibrous adhesive peritonitis between liver, mesentery and stomach. The individual was also heavily infested with *Ascaris suum*. Again, the evisceration appeared simpler than if this carcass was presented on a conventional line. The evisceration could also be undertaken hygienically as the whole set could be removed before further examination.

The research group strives to provide the meat industry with a robust, flexible and scalable cognitive robotic platform [8]. The removal of an entire set of internal organs appeared feasible for an automated evisceration procedure because the robot may need only one gripping point (trachea) and one relatively simple operation, compared to delicate identification, gripping and removal of the pluck or specific internal organs.

MFC is more robust against downtime because an automated conventional line stops if a machine in the line is out of order. MFC is more flexible because the processing can adjust to varying sizes of animals without reducing the speed in neighbouring cells, or development and maintenance can be undertaken in one cell without interfering with the others. A factory can operate an optimal number of cells depending on day to day variation. Working from outside in, makes it possible to apply cheaper cognitive elements (sensors, cameras and artificial intelligence), e.g. 3D cameras to control movements and optimise cutting trajectories by artificial intelligence. Alternatively, conventional automated lines apply many and expensive specialised machines with low flexibility, e.g. Danish Crown’s plant in Horsens, Denmark [9]. Another example of an automation solution in cold meat cutting depends on control information obtained from expensive x-ray systems that produce a static picture from one point in time before processing, not able to adapt to the configural changes when a carcass is processed warm [10].

However, from a meat technological perspective there are several pro and cons. The hams, with the present cutting trajectories, are leaving the hip bone attached to the spine and back part, resulting in a cut that, for example, deviates from the Spanish dry-cured ham raw material. On the positive side, meat producers could customise the cooling regimes for specific cuts and improved water binding effects of warm pre-salted trimmings.

A disadvantage was that the set of internal organs needs to be handled afterwards to separate parts and organs to present them for meat inspection. This was not studied as customised equipment needs to be constructed for these operations. It could also be a challenge to rip off the flare fat in an entire piece.

The two pigs with lesions became examples where the evisceration in case of pathology could be performed more hygienically with the MFC approach with reduced probability of accidental contamination of the carcass from ruptures.

In conclusion, Meat Factory Cell evisceration can be undertaken without the need to cut through the gastrointestinal tract. It is a novel procedure reducing the probability of accidental faecal contamination from gut content of pork carcasses, and we also think it will reduce the complexity of the evisceration procedure and therefore be suitable for automated evisceration.

## Data Availability

Data sharing not applicable to this article as no datasets were generated or analysed during the current study.

## Abbreviations

CHU: Carcass Holding Unit
MFC: Meat Factory Cell
